# Extracellular Volume by Computed Tomography Is Useful for Prediction of Prognosis in Dilated Cardiomyopathy Cases with Heart Failure with Reduced Ejection Fraction

**DOI:** 10.3390/jcdd11120399

**Published:** 2024-12-11

**Authors:** Satomi Yashima, Hiroyuki Takaoka, Joji Ota, Moe Matsumoto, Yusei Nishikawa, Yoshitada Noguchi, Shuhei Aoki, Kazuki Yoshida, Katsuya Suzuki, Makiko Kinoshita, Haruka Sasaki, Noriko Suzuki-Eguchi, Tomonori Kanaeda, Yoshio Kobayashi

**Affiliations:** 1 Department of Cardiovascular Medicine, Chiba University Graduate School of Medicine, Chiba 260-8677, Japan; 2 Department of Radiology, Chiba University Hospital, Chiba 260-8677, Japan; 3 Department of Cardiology, Eastern Chiba Medical Center, Togane 283-0000, Japan

**Keywords:** cardiomyopathy, fibrosis, prognosis

## Abstract

Objective: Cardiac computed tomography (CT) helps screen coronary artery stenosis in cases with dilated cardiomyopathy (DCM). Extracellular volume fraction (ECV) analysis has recently been eligible for CT. Method: We evaluated the impact of ECV on the CT to predict the prognosis in DCM patients with heart failure with reduced ejection fraction (HFrEF). Patients or Materials: We analyzed 101 consecutive DCM cases with HFrEF who underwent cardiac CT. All the patients had a lower left ventricular (LV) ejection fraction (LVEF) of less than 40%. We evaluated the effect of ECV to predict the patients’ prognosis. Cardiovascular death, hospitalization due to heart failure, and fatal arrhythmic events were included in the major adverse cardiac events (MACE). Results: MACE occurred in 27 cases (27%). The patients with MACE (27 cases) had an increased ECV on the LVM on the CT (37.2 ± 6.7 vs. 32.2 ± 3.6%, *p* = 0.0008) compared to the others (74 cases). Based on the receiver operating characteristics curve analysis, the best cutoff value of the ECV on the LVM to predict the MACE was 32.3%. The patients with ECV ≥ 32.3% had significantly higher MACE based on the Kaplan–Meier analysis. The ECV on the LVM was a significant marker to predict MACE based on the univariate Cox proportional hazard model (hazard ratio of 8.00, 95% confidence interval 1.88–33.97, *p* = 0.0048). Conclusions: ECV by CT is helpful to predict MACE in cases with DCM and HFrEF.

## 1. Introduction

Cardiac computed tomography (CT) has been helpful as a screening test for cases suspected of coronary artery stenosis [1,2]. In recent years, technological advancements in CT, including multi-detector scanners and new image reconstruction methods, have further improved the diagnostic accuracy of coronary artery stenosis, leading to its widespread use in clinical practice [3]. Additionally, with improved image quality and reduced radiation exposure during CT, there is growing interest in assessing myocardial characteristics using delayed contrast-enhanced imaging [4].

Dilated cardiomyopathy (DCM) is a specific cardiomyopathy with left ventricular (LV) dilatation and abnormal function, resulting in idiopathic low cardiac output unrelated to conditions such as hypertension, valvular disease, or coronary artery disease [5]. It is known for its chronic heart failure symptoms and a clinical course marked by recurrent acute exacerbations, with a prognosis that can be poor and progressive. However, recent developments in heart failure therapies have improved outcomes, and early intervention can lead to a better prognosis in some cases [6]. DCM is relatively common in routine cardiovascular practice, and according to 2022 statistics on heart transplants, it accounts for approximately 64% (362 cases out of 565 cases) of adult heart transplantations due to primary diseases [7]. 

In dilated cardiomyopathy, the prognostic factors have been known to be cardiac dysfunction, cardiac enlargement, and the presence of late enhancement in MRI [8,9]. ECV is a quantitative measurement used in cardiac imaging, particularly in cardiac MRI (magnetic resonance imaging), to assess the proportion of the heart muscle that is made up of the extracellular matrix (outside the cells) rather than the cells themselves. ECV reflects the amount of fibrosis or scarring in the myocardium, which can occur in various heart diseases such as dilated cardiomyopathy (DCM), hypertrophic cardiomyopathy, and other forms of cardiomyopathies [10,11]. In the cases of DCM, there have been reports that a higher ECV on an MRI means myocardial fibrosis, suggesting that this parameter can serve as a prognostic factor for DCM [10,11]. In addition, the studies of DCM cases have indicated that ECV values are useful for predicting LV reverse remodeling [12].

While the clinical utility of MRI-based ECV analysis has been demonstrated in previous studies, MRI has limitations, including contraindications and imaging conditions, and the number of examinations remains relatively low [13]. We are considering the increasing number of heart failure patients and the demand for cardiac MRI in recent years. However, cardiac MRI is challenging to increase in volume due to factors such as the time-consuming nature of the imaging process, several contraindications, and the requirement for skilled technicians. There is also competition with multi-organ imaging. Furthermore, imaging is problematic in arrhythmia or when breath-holding is challenging, and the image quality is prone to deterioration. 

On the other hand, cardiac CT has been performed in many cases with DCM, primarily to evaluate coronary artery stenosis. The diagnosis of DCM requires the exclusion of coronary artery disease, and cardiac CT, which can be performed in a wide range of cases, and has been invaluable as a screening test [14]. With advancements in CT equipment and image analysis software, it has become possible to perform delayed contrast-enhanced imaging after an early contrast-enhanced scan for coronary artery evaluation, the visual assessment of the late enhancement of LV myocardium (LVM), and ECV analysis has been eligible [15]. Given these advancements, we previously reported on the usefulness of assessing ECV with CT in predicting the prognosis of DCM [16]. This time, we strictly limited our study to cases of HFrEF within DCM and increased the number of cases for a similar investigation, which we now report the result of. The prognostic value of an additional left ventricular delaying the contrast imaging in cardiac CT for screening coronary artery disease has not been established in patients with DCM with HEFrEF, and this study will prove it.

## 2. Materials and Methods

### 2.1. Patient Selection

We evaluated the ECV in the LVM on the CT in 101 DCM patients with reduced LVEF (LVEF < 40%) who underwent cardiac CT, including non-contrast and late-phase scanning using the same tube voltage, from January 2009 to September 2023 at Chiba University Hospital (n = 89) or from July 2015 to December 2022 at Eastern Chiba Medical Center (n = 12) and investigated their association with major adverse events (MACE). The patients with other cardiomyopathies or primary valvular diseases or significant coronary artery disease were excluded, as were previous similar studies [10,11,16]. 

We defined significant coronary artery stenosis as a > 70% stenosis (>50% stenosis only in left main coronary artery) on cardiac CT [17]. A composite of cardiovascular death, hospitalization due to heart failure and fatal arrhythmic events were defined as MACE. Follow-up data were extracted from the patient’s medical records at our institutions. Patient background information, including coronary artery disease risks and treatment, was also obtained. This retrospective analysis received approval from the institutional review board (reference number 3822).

### 2.2. The Protocol of Computed Tomography

CT scans were conducted using either a 256-slice CT scanner (Revolution CT Apex, GE Healthcare, Waukesha, WI, USA) or a 320-slice CT scanner (Aquilion One or Aquilion One/ViSION Edition, Canon Medical Systems, Otawara, Japan), with the patients positioned in the supine position. A non-contrast prospective ECG-gated cardiac scan was performed following a scout scan. The slice thickness and tube voltage were set at 0.625 mm and 70 kV for the 256-slice CT, and 0.5 mm and 80–120 kV for the 320-slice CT, respectively.

In the cases where retrospective ECG gating was used, the dose modulation technique was applied, when possible. Conventional contrast-enhanced CT scans were conducted with a tube voltage and slice thickness of 120 kV and 0.625 mm for the 256-slice CT, and 120 or 135 kV and 0.5 mm for the 320-slice CT, respectively [18]. The tube current was determined by an automatic exposure control system. The patients with higher heart beats of ≥65 beats per minute took either 12.5 mg of landiolol or 20 mg of metoprolol before scanning, unless contraindicated for beta-blockers. All the patients were administered two doses of sublingual isosorbide dinitrate just before the early-phase contrast scan.

For contrast injection, a standard triphasic protocol was applied, as previously shown [16]. A dual-syringe injector with a dual-flow feature (Dual Shot, Nemoto, Tokyo, Japan) was used for the intravenous contrast injection. Forty-five to 100 mL of undiluted iodinated contrast media (350–370 mg/mL) was injected at 3–5 mL/s at the beginning, followed by 0–70 mL of a 50%/50% saline-to-contrast mixture at 2–4 mL/s, and finally, 16–40 mL of pure saline at 2–4 mL/s.

A prospective ECG-gating scan was performed six minutes after the administration of iodine contrast for a late-phase scan [16]. The CT scans were performed with the slice thickness and tube voltage, which were set as in the non-contrast scan.

### 2.3. Analysis of ECV on CT

**Figure 1:**
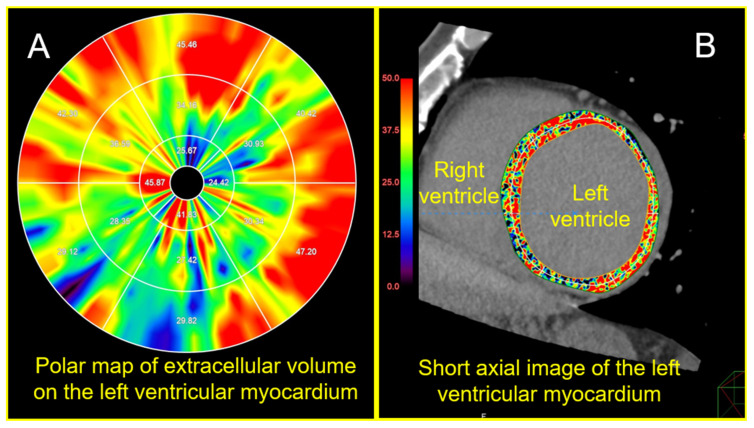
Left ventricular extracellular volume (ECV) analysis image.

A clinically available image analysis software (Ziostation 2, Ziosoft Inc., Tokyo, Japan) was used for the measurement of the ECV on the CT with the following formula: ECV = (ΔHUm/ΔHUb) (1 − Hct), where ΔHUm represents the change in myocardial CT attenuation in the Hounsfield units (HU), ΔHUb represents the change in the CT attenuation of the blood, and Hct is the hematocrit value [19]. Three-dimensional non-rigid registration of the myocardium between the late-phase and the non-contrast images was performed by the software to make subtraction images [19]. The subtraction images were used to calculate the change in the CT attenuation (ΔHU). A polar map that showed the mean ECV value for each of the 16 LVM segments, which was defined by the American Heart Association, was generated by the software (Figure 1A,B).

### 2.4. The Protocol and Analysis on TTE

The EPIQ system and X5-1 transducers (Philips Medical Systems, Andover, MA, USA) or Vivid E9 and M5S transducers (GE Vingmed, Horten, Norway) were used for the analysis of TTE as routine clinical practice by the standard methods according to the latest guidelines [20]. The LV volumes and systolic ejection fraction (LVEF) were evaluated using the modified Simpson method in the apical view. The E/e’ was also measured to evaluate the diastolic function.

Presented here are the LV ECV analysis images obtained from a cardiac CT scan of a 59-year-old male diagnosed with dilated cardiomyopathy. The patient experienced ventricular fibrillation two months after the cardiac CT imaging. The average LV ECV was elevated, measuring 34.2%.

A cardiologist with five years of experience in the analysis of cardiac CT (SY) calculated the ECV of the LVM, and these values were used for subsequent analysis. Additionally, another board-certified cardiologist with 14 years of experience in the analysis of cardiac CT (HT) independently performed the ECV analysis, and the inter-observer agreement was evaluated.

**Figure 2:**
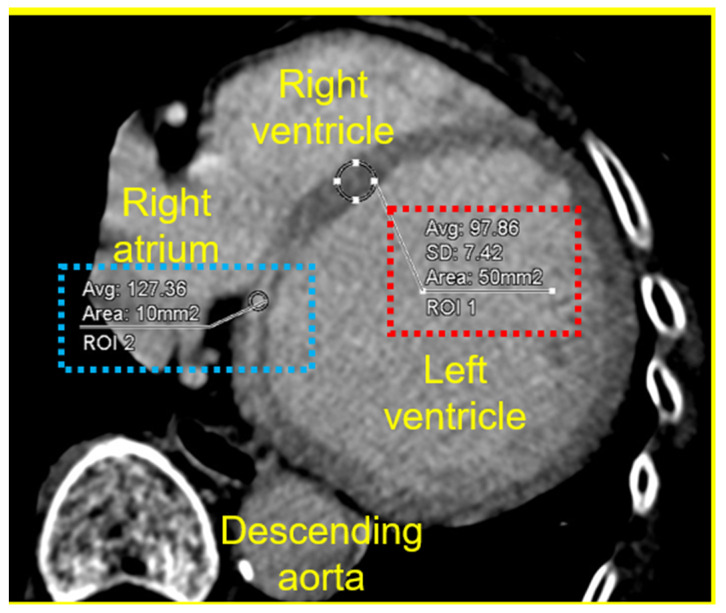
Details of the measurement of contrast noise ratio in late-phase computed tomography images.

The image quality of the late-phase scan was quantitatively analyzed. First, approximately 10-mm^2^ regions of interest (ROIs) on the LE (Figure 2, blue square) and approximately 50-mm^2^ ROI on the remote normal LVM without LE were selected (Figure 2, red square). The ROIs were manually drawn for the patients with LE or the remote normal segment of the LVM on CT, and the CT attenuation values were measured.

We also quantified the contrast-to-noise ratio (CNR), defined as the difference in attenuation between the LE and the remote normal LVM divided by the standard deviation (SD) of the remote normal LVM attenuation [21]. The CNR analysis and visual assessment of the LE on the CT was performed by a cardiologist (SA), using late phase CT images which were reconstructed at 5 mm slice thickness.

### 2.5. Statistical Analysis

For the continuous variables, the results were reported as either the mean ± standard deviation (SD) or the median with interquartile range (IQR), depending on the data distribution. The categorical variables were reported as absolute numbers and percentages. Comparisons of the continuous variables were performed using Student’s *t*-test, while the categorical variables were analyzed with Fisher’s exact test. The agreement between the two observers’ evaluations of the ECV of the LVM on the CT was assessed using the correlation coefficient. All the statistical tests were two-tailed, with a significance threshold set at *p* < 0.05.

The receiver operating characteristic (ROC) curve analysis was conducted to evaluate the association with MACE, and the optimal cutoff value was identified using the Youden index. Time-to-event data, including MACE occurrence and rates, were analyzed using Kaplan–Meier methods, with the group differences assessed by the log-rank test. To investigate the relationship between the predictor variables and the time to the MACE, a Cox proportional hazards model was employed. JMP version 15.0.0 (SAS Institute Inc., Cary, NC, USA) was used for statistical analyses.

## 3. Results

Among the 101 cases, 27 cases (27%) experienced MACE (two cardiac death, five fatal arrhythmias, 20 hospitalizations for heart failure) during the observation period (the follow-up period after CT: 40 ± 46 months) (Table 1). 

No significant difference in background between the patients with MACE and those without (Table 2) was detected. The serun BNP was measured only in 68 patients (67%).

The patients with MACE (27 cases) had an increased LV ECV (%) on their cardiac CT (37.2 ± 6.7 vs. 32.2 ± 3.6%, *p* = 0.0008) compared to the other cases (74 cases) (Table 3). The E/e’ of the LVM on the TTE was measured in 95 patients (94%).

There was no significant difference in the presence of LE in the LVM on the CT between the two groups, but the patients with MACE (27 cases) had a higher prevalence of epicardial LE in the LVM on their CT than the patients without MACE (15% vs. 3%, *p* = 0.031).

**Figure 3:**
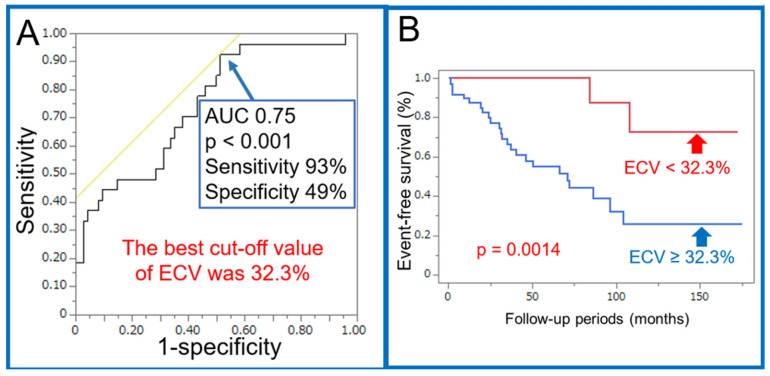
Receiver operating characteristics (ROCs) analysis of left ventricular (LV) extracellular volume (ECV) (**A**) and Kaplan–Meier analysis based on LV ECV on CT imaging for predicting adverse cardiac events (**B**) in cases of dilated cardiomyopathy with impaired left ventricular systolic function on CT Imaging.

A ROC analysis revealed an optimal threshold of 32.3% for ECV in predicting adverse cardiac events (AUC = 0.75, *p* < 0.001, sensitivity 93%, specificity 49%) (Figure 3A). A Kaplan–Meier analysis showed that the group with ECV > 32.3% had significantly lower survival rates than the group with ECV ≤ 32.3% (*p* < 0.01) (Figure 3B).

A receiver operating characteristics (ROCs) analysis was performed, and the optimal threshold for the LV ECV was 32.3% to predict major adverse cardiac events. The Area Under the Curve (AUC) was 0.75 (*p* < 0.001) (A). The Kaplan–Meier analysis results indicated a significantly lower survival rate in the group with LV ECV ≥ 32.3% (27 cases) compared to the group with ECV < 32.3% (74 cases) (*p* < 0.01) (B).

The group with ECV > 32.3% had a significantly larger LVEDV than the group with ECV ≤ 32.3% (*p* < 0.01) (Table 4). The serun BNP was measured only in 68 patients (67%). The E/e’ of the LVM on the TTE was measured in 95 patients (94%).

A Univariate Cox proportional hazard analysis showed that only an ECV > 32.3% on the CT was a significant prognostic factor (hazard ratio 8.0, 95% confidence interval 1.88–33.97, *p* = 0.0048) (Table 5). The ECV on the LVM was negatively correlated with the LVEF on the TTE (Pearson’s correlation coefficient was −0.11, *p* = 0.27). The ECV on the LVM was positively correlated with the E/e’ on the TTE (Pearson’s correlation coefficient was 0.26, *p* = 0.012).

The CNR was 4.1 ± 1.1 in 30 cases (30%) with LE. The computed tomography dose index (CTDI) for the late-phase scan in our population was 15.4 ± 5.0 mGy in 89 patients (the radiation dose report was not available in 12 patients). The inter-observer consistency of the ECV of the LVM on the CT was 0.85.

## 4. Discussion

The findings of this research propose that the assessment of LVM’s ECV on CT could serve as a potential predictor of future MACE occurrences in patients with DCM and HFrEF. CT plays a valuable role in identifying coronary artery stenosis, and when accompanied by an additional late-phase scan, it becomes feasible to perform ECV analysis. These results indicate that CT may serve as a valuable modality for comprehensive cardiac screening in patients with DCM and HFrEF.

### 4.1. ECV Analysis on MRI in DCM Cases

The late gadolinium enhancement (LGE) of the LVM on the MRI is a sensitive marker of the future cardiac events in DCM patients, as myocardial abnormality is closely associated with reduced cardiac function and the development of lethal arrhythmias [9]. However, it is important to note that nearly two-thirds of DCM patients do not exhibit LGE, as LGE evaluation is primarily qualitative and may not effectively detect diffuse myocardial fibrosis. Recently, the use of T1 mapping on MRI has emerged as a method to predict future MACE [7].

MRI may be contraindicated in some DCM cases due to factors such as the presence of claustrophobia or implanted mechanical devices. Additionally, gadolinium contrast may not be suitable for patients with renal dysfunction [9]. Third, in the presence of arrhythmias such as atrial fibrillation or ventricular premature beats in DCM cases, or in cases where breath-holding is difficult due to dyspnea caused by heart failure, the image quality may be reduced by artifacts and a longer imaging time may be required. In such conditions, the highly specialized skills of radiology technologists are crucial for obtaining high-quality images. Furthermore, since T1 mapping is a relatively new cardiac MRI sequence, it may only be available on modern scanners in well-equipped facilities. In contrast, cardiac CT, including late-phase scans, can be performed quickly without significant challenges even in patients with arrhythmia, as each scan has a short duration without long breath-holding. Moreover, CT is more accessible than MRI, with nearly twice as many CT machines as MRIs in our country [22]. 

Increased ECV in the LVM generally corresponds to a greater amount of myocardial fibrosis, which is detected on myocardial biopsy [23]. Consequently, a higher ECV signifies the severe degeneration of LVM, which is associated with reduced LV function and an increased risk of ventricular arrhythmic events [8]. Myocardial fibrosis has been identified as a significant parameter for predicting patient prognosis in several myocardial conditions [5,6].

### 4.2. Clinical Implication of ECV on CT

ECV in the LVM means the extent of myocardial fibrosis, as previously discussed [23], and the ECV value is consequently more stable than the TTE-derived parameters. The patients with preserved LVEF showed significant relationships between increasing the ECV and the reduced systolic and diastolic radial and long-axis peak velocities [24]. In fact, the ECV in the LVM was negatively correlated with the LVEF as assessed by the TTE in the present study. The ECV on the CT of the severe aortic stenosis cases before invasive treatment is known to be useful for predicting postoperative prognosis [25]. In addition, the studies of DCM cases have indicated that ECV values are useful for predicting LV reverse remodeling [12]. Additionally, it has been demonstrated that ECV analysis using preoperative CT helps predict the recovery of cardiac function after ablation in patients with atrial fibrillation and reduced cardiac function [26]. Furthermore, the ECV in the LVM is elevated in hypertensive heart disease (HHD) patients, particularly in those with LV systolic dysfunction [27]. Therefore, several kinds of cardiac abnormalities have a significant influence on the degree of the ECV of the LVM on the CT. Notably, the increase in the ECV in the LVM can be observed in the pre-disease stage of DCM, highlighting its sensitivity as compared to LVEF [2].

The present study showed that patients with a high ECV have a high risk of future events, which may serve as a guide for the aggressive introduction of cardioprotective drugs and device therapy such as CRT for such patients.

### 4.3. Competitive Risk Factors in DCM Patients

This study considered several clinical factors as potential risks for DCM patients. The ECV in the LVM and the presence of epicardial LE in the LVM on the CT were considered possible risk factors of MACE. However, only the ECV on the CT emerged as an independent risk factor for future cardiac events in our DCM patients. TTE findings, such as the degree of valvular abnormalities or the size of the cardiac chamber, were easily influenced by various factors, such as the condition of heart failure, the presence of arrhythmias, and the patient physique; therefore, the effect of the TTE parameters on the patient prognosis was weaker than the ECV of LVM. 

It is important to highlight that the percentage of epicardial LE in LVM on CT is significantly higher in patients with MACE than those without. Still, overall, the LE was not a significant risk factor, contrasting with the findings of the previous MRI study [9]. This discrepancy may be attributed to LE’s relatively lower image quality on CT, which may impede visual assessment. Furthermore, due to the low contrast, the visual evaluation of LE on LVM requires expertise. However, ECV analysis can be quickly evaluated even by examiners unfamiliar with interpreting cardiac CT, as it utilizes existing image analysis software. Therefore, ECV analysis remains a suitable alternative and has demonstrated its ability to provide clinically significant information in DCM. 

### 4.4. Image Quality of Late Enhancement on CT

It is important to note that the contrast resolution of MRI is superior to CT; therefore, cardiac MRI is the reference standard to detect myocardial fibrosis. CT attenuation values increase when using a lower tube voltage, but image noise also increases because of limited radiation exposure; therefore, it has been challenging to apply to late-phase scanning [28]. However, higher maximum tube currents and newer reconstruction techniques in CT scanners have emerged, which help reduce image noise and the effective radiation dose in lower tube voltage images. Our previous research has shown that a combination of state-of-the-art CT technology and iterative reconstruction techniques is effective in improving the image quality for the LE on CT and enhancing diagnostic accuracy to detect them [4]. Additionally, ECV analysis provides quantitative data and visualizes it with a color map. This makes detecting the LE in the LVM on CT visually relatively easy, which traditionally required skilled visual assessment. Significantly, this improvement in image quality for the LE in the LVM has led to the recommendation of a late-phase scan of CT as an alternative to MRI, if it is not available, in the latest guidelines for cardiac amyloidosis from the Japanese Cardiovascular Society and for cardiac CT [29,30].

For the performing diagnosis of the LE of the LVM on CT, it is important to ensure that the image quality is sufficiently maintained. The CNR for the 30 patients with LE in LVM on CT was 4.1 ± 1.1 in this study. The CNR values for the LE on CT in the old myocardial infarction patients were around six and two in two previous pieces of research, and the CNR value for the LE in our research was similar, despite differences in the background myocardial disease [19,21].

### 4.5. Additional Radiation Exposure for Extracellular Volume Analysis on CT

ECV measurement requires additional radiation exposure than usual, but this is a trade-off for valuable clinical data. The computed tomography dose index (CTDI) for the late-phase scan in our population was 15.4 ± 5.0 mGy. Wide coverage multi-detector CTs and advances in new image reconstruction techniques have contributed to a reduction in the radiation dose for late-phase cardiac imaging, simultaneously improving image quality [4]. These developments have mitigated the drawbacks associated with additional late-phase cardiac imaging, making it advisable for DCM cases. With the upcoming introduction of photon-counting CT, further improvements in image quality and reductions in radiation exposure are anticipated. As CT diagnostics are expected to expand, the results of this study are expected to become highly valuable as important initial data [31].

### 4.6. Limitations of This Study

This study has several limitations. Firstly, this study was conducted retrospectively at bi-centers; therefore, the sample size of this research was relatively small and a larger prospective study is needed to prove the impact of the results of the present study. Secondly, a single-energy scan was applied for the ECV analysis, necessitating the subtraction of the late-phase and non-contrast images, which might cause the ECV analysis to be incorrect because of the misregistration of the two data sets. 

We included the cases diagnosed mainly based on echocardiography and cardiac CT findings, and myocardial biopsy was not performed in all the cases. We excluded the patients who obviously had the other non-ischemic etiologies of low LV function, but it cannot be ruled out that there may be cases with cardiomyopathies of other etiologies. Additionally, cardiac CT was only performed in the cases suspected of coronary artery disease. Therefore, the selection bias of the patients with DCM might affect the result. Finally, this study included cases that had undergone CT around 2010, so it was difficult to examine the efficacy of newer heart failure drugs such as angiotensin receptor/neprilysin inhibitors in combination.

## 5. Conclusions

ECV by CT is useful to predict patient prognosis in cases with DCM and HFrEF.

CT was originally a modality focused on coronary artery assessment, but it was found to be useful in detecting myocardial damage and predicting prognosis, and is expected to be further utilized in clinical practice in the future.

## Figures and Tables

**Table 1:** Patient background.

		n = 101
Age, years		57.5 ± 13.9
Male, n (%)		74 (73)
Hypertension, n (%)		38 (38)
Dyslipidemia, n (%)		24 (24)
Diabetes Mellitus, n (%)		20 (20)
Atrial fibrillation, n (%)		19 (19)
Medications	β-blocker, n (%)	69 (68)
	Angiotensin Receptor II Blocker (ARB), n (%)	30 (30)
	Angiotensin-Converting Enzyme Inhibitor (ACE-i), n (%)	30 (30)
	Mineralocorticoid Receptor Antagonists (MRB) (%)	51 (50)
	Statin, n (%)	23 (23)
	Sodium Glucose Cotransporter 2 (SGLT2), n (%)	7 (7)

**Table 2:** Comparison of background between the patients with MACE and without.

		MACE (+) (n = 27)	MACE (−) (n = 74)	*p*-Value
Age, years		56 ± 12	58 ± 15	0.64
Male, n (%)		22 (81)	52 (70)	0.26
Hypertension, n (%)		10 (37)	28 (38)	0.94
Dyslipidemia, n (%)		7 (26)	17 (23)	0.76
Diabetes Mellitus, n (%)		3 (11)	17 (23)	0.18
Atrial fibrillation, n (%)		5 (19)	14 (19)	0.96
Serum BNP level (pg/mL)		538 ± 583	393 ± 564	0.32
Medications	β-blocker, n (%)	17 (63)	52 (70)	0.80
	statin, n (%)	7 (26)	16 (22)	0.65
	ACE-i or ARB, n (%)	17 (71)	28 (55)	0.22
	MRB, n (%)	17 (63)	34 (46)	0.13
	SGLT2, n (%)	0 (0)	7 (9)	0.18
Follow-up period (months)		41 ± 34	40 ± 50	0.88

**Table 3:** Comparison of the TTE and CT findings between the patients with and without MACE.

	MACE (+) (n = 27)	MACE (−) (n = 74)	*p*-Value
LVEF on TTE (%)	25 ± 9	28 ± 8	0.147
End Diastolic Volume (EDV) index on TTE (mL/m^2^)	127 ± 60	112 ± 42	0.280
End Systolic Volume (ESV) index on TTE (mL/m^2^)	51 ± 39	38 ± 31	0.282
E/e’ of LVM on TTE	15.6 ± 7.2	13.2 ± 5.7	0.143
ECV of LVM on CT (%)	37.2 ± 6.7	32.2 ± 3.6	0.0008
Late enhancement on LVM on CT, n (%)	12 (44%)	18 (24%)	0.0502
Septum mid, n (%)	6 (23%)	9 (12%)	0.180
Epicardium, n (%)	4 (15%)	2 (3%)	0.031
Endocardium, n (%)	0 (0%)	0 (0%)	-
Overlap, n (%)	1 (4%)	7 (9%)	0.36

**Table 4:** Comparison of patient background between the patients with LV-ECV ≥ 32.3% and without.

		LV-ECV < 32.3% (N = 38)	LV-ECV ≥ 32.3% (N = 63)	*p*-Value
Age, years		58 ± 15	57 ± 13	0.87
Male, n (%)		25 (66)	49 (78)	0.19
Hypertension, n (%)		16 (42)	22 (35)	0.47
Dyslipidemia, n (%)		8 (21)	16 (25)	0.62
Diabetes Mellitus, n (%)		8 (22)	12 (19)	0.86
Atrial fibrillation, n (%)		9 (24)	10 (16)	0.33
Serum BNP level (pg/mL)		486 ± 268	596 ± 382	0.14
Medications	β-blocker, n (%)	30 (79)	39 (62)	0.075
	statin, n (%)	5 (13)	18 (29)	0.074
	ACE-i or ARB, n (%)	22 (59)	37 (59)	0.93
	MRB, n (%)	15 (39)	36 (57)	0.085
	SGLT2, n (%)	2 (5)	5 (8)	0.70
Follow-up period (months)		49 ± 39	44 ± 34	0.88
LVEF on TTE (%)		29 ± 9	26 ± 8	0.081
End Diastolic Volume (EDV) Index on TTE (mL/m^2^)		36 ± 29	52 ± 41	0.021
End Systolic Volume (ESV) Index on TTE (mL/m^2^)		34 ± 27	45 ± 36	0.028
E/e’ of LVM on TTE		12.8 ± 5.5	14.5 ± 6.6	0.17
Late enhancement on LVM on CT, n (%)		7 (18%)	23 (37%)	0.054

**Table 5:** A Univariate Cox proportional hazard analysis.

	Uni-Variate Analysis		
	Hazard Ratio	95% Confidence Interval	*p* Value
Epicardial late enhancement on LVM on CT, n (%)	1.53	0.53–4.48	0.43
ECV of LVM on CT ≥ 32.3%	8.00	1.88–33.97	0.0048

## Data Availability

No data will be shared related to this study.
